# Phytolacca Decandra as a Remedial Agent in Mastitis

**Published:** 1879-11-20

**Authors:** Wm. F. Holt

**Affiliations:** Macon


### *PHYTOLACCA DECANDRA AS A REMEDIAL AGENT IN MASTITIS.*

BY WM. F. HOLT, M. D., MACON.

The accoucheur who has safely conducted his patient through the travails of labor, perhaps tedious and complicated; who witnessed the maternal emotions of the fond parent when the cry of the infant caused her whole frame to vibrate with a thrill of joy, since it unmistakably announced to her that the dream of her young life was fondly realized, should feel profoundly grateful that through his agency the safety of both mother and child are assured.

But, having congratulated himself that "all is well," yet he well knows that dangers may arise that will tax to the utmost his professional skill. His mind dwells upon the various diseases incident to the parturient chamber. Puerperal fever and puerperal peritonitis loom up before him and fill him with anxious forebodings. Mastitis and mammary abscess, with its tedious suppuration, hectic fever and attendant sufferings, while not presenting so formidable an array of symptoms, or so destructive to life, yet causes him anxious solicitude, since the treatment hitherto adopted has been so unsuccessful. Until within a few years it was a problem—one that has not been successfully demonstrated, as to what was the best method of treating mastitis. Divers remedies had been suggested, various specifics announced and heralded to the profession as the remedy "par excellence;" all in turn have been faithfully tried and consigned to merited oblivion.

So often had the profession been disappointed in the various remedial agents suggested for the successful treatment of this obstinate affection, that it came to be regarded as almost the opprobrium of the profession.

An old and experienced physician—one who enjoyed a large and lucrative practice—who stood in the front rank of his profession, justly enjoying the confidence of his numerous patrons, once remarked to the writer (who had consulted him as to the best means to be employed in the treatment of mastitis) that he knew of no method that positively promised good results. That he would suggest as soon as the gland became hard and painful, that hot formentations or poultices be constantly and assiduously applied (thereby inviting suppuration) until pus had formed, and then to lance freely; that, in his judgment, it was not only the best method of dealing with it, but one that caused less distress to the patient. Not feeling satisfied, I in turn tried various remedies that were offered, and finally concluded that we had in belladonna locally applied a good agent, but not one that met all the requirements.

Within a few years my attention was directed to the phytolacca decandra as a remedy that met all the indications. I at once resolved to give it a fair test. Soon an opportunity presented itself. On the night of November 12, 1876, I was called to see Mrs. A. B., in labor with her third child, Nothing unusual occurred, and on the following morning the labor successsully terminated. On visiting her the third day my attention was directed to the hard and painful breast; her mind was filled with gloomy forebodings, as she informed me that in both of her previous confinements they had caused her intense suffering and had to be lanced several times, and her convalescence was slow and tedious. Upon examination I found both glands filled with several hard nodules and very painful. She stated that previously, she had used various plasters that had been recommended, and that belladonna, both in the form of ointment and the fluid extract, had been used. I at once ordered her to take five drops of the fluid extract of phytolacca decandra every three hours until its constitutional effect was produced, and then suspend its use or lengthen the intervals. I confess that my faith in the remedy was not very great, but it was the only one at my command. She faithfully carried out my directions, and in a few days all traces of the inflammation had disappeared. The same lady was confined again in 1878; the same symptoms presented themselves, and were again promptly relieved by the phytolacca decandra administered as before. I could produce several more cases from my note book, showing the good effect of the remedy, but as all were attended with similar results, deem it unnecessary.

My friend, Dr. Charles H. Hall, of this city, who regards the phytolacca as the only remedy in mastitis, informs me that he uses the fluid extract as a local application to the gland in addition to giving it internally, and that it rarely disappoints him in arresting the inflammation.

In this connection I would remark that the most reliable preparation I have used is the fluid extract (from the fresh root) prepared by W. S. Merrell & Co., of Cincinnati, Ohio. While I am aware, Mr. President, that this is not a new remedy, yet it is one that has been so successful in my hands that I felt warranted in directing the attention of the profession to it, feeling assured that he who tries it faithfully, and uses a reliable preparation of the fresh root, will find that nothing more is desired, and that this troublesome and painful affection can almost with certainty be averted.—Transactions of the Medical Association of Georgia.

